# Unilateral Pompholyx in a Patient of Anterior Horn Disease: An Unusual Presentation

**DOI:** 10.5826/dpc.1103a47

**Published:** 2021-07-08

**Authors:** Biswanath Behera, Rashmi Kumari, Debasis Gochhait, Pavithra Ayyanar

**Affiliations:** 1 Department of Dermatology, and Venereology, AIIMS, Bhubaneswar, India; 2 Department of Dermatology, Venereology and Leprology, Jawaharlal Institute of Postgraduate Medical Education and Research (JIPMER), Puducherry, India; 3 Department of Pathology, Jawaharlal Institute of Postgraduate Medical Education and Research (JIPMER), Puducherry, India; 4 Department of Pathology, AIIMS, Bhubaneswar, India

**Keywords:** Dermoscopy, eczema, dyshidrotic, pompholyx

## Introduction

Pompholyx is a type of eczematous dermatitis, presenting deep-seated vesicles and bullae (blisters) over the bilateral acral skin. Isolated palmar involvement is the most common (70%), followed by palmoplantar (20%), and isolated plantar involvement (10%). Diagnosis is rarely difficult when it presents in the classical form, although, at times, dermatoses such as dyshidrosiform bullous pemphigoid, epidermolysis bullosa aquisita, and in the pediatric population, scabies and acropustulosis need to be ruled out. The unilateral localization of pompholyx is an unusual presentation [1].

## Case Presentation

**Figure 1:**
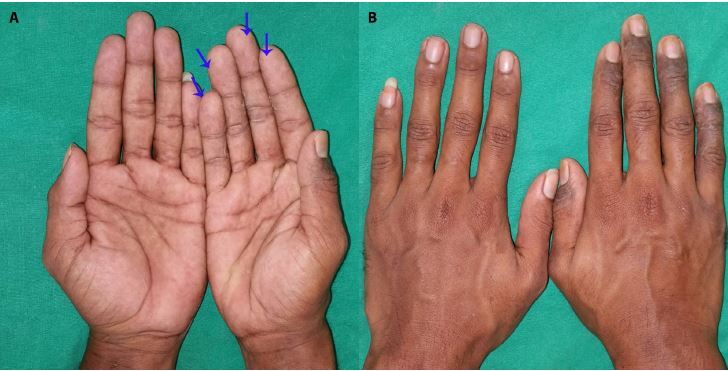
(A) and (B) Multiple deep-seated vesicles (arrows) over the palmar, lateral, and dorsal aspect of right medial four digits, along with areas of desquamation. The contralateral hand is within normal limits.

**Figure 2:**
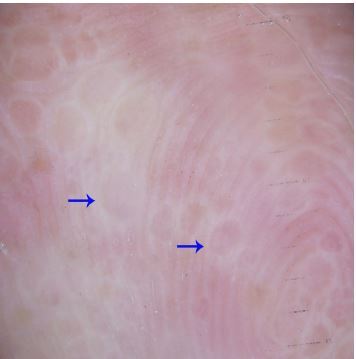
Dermoscopy under nonpolarized mode showing featureless areas.

**Figure 3:**
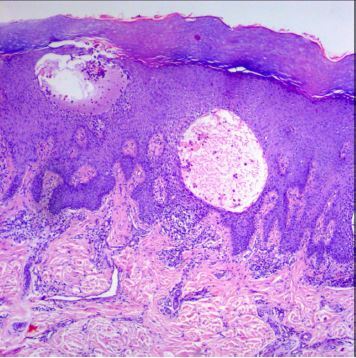
Histology showing the acanthotic epidermis, spongiosis, spongiotic vesicles, and upper perivascular mild lymphocytic infiltration (H&E, ×100).

A 25-year-old male had a 2-months history of itchy lesions over the right hand, associated with increased sweating. The patient was a known case of anterior horn disease and had right upper limb paresis. He denied any personal or family history of atopy, prior history of contact dermatitis, or adverse drug reaction. Cutaneous examination showed multiple deep-seated vesicles over the palmar, lateral, and dorsal areas of the right medial 4 digits, with areas showing desquamation (Figure 1). The contralateral hand was within normal limits. Upon nonpolarized dermoscopy only featureless areas (Figure 2) were evident. Therefore, a differential diagnosis of pompholyx and tinea manuum was considered. Histological analysis showed acanthotic epidermis, spongiosis, spongiotic vesicles, and upper perivascular mild lymphocytic infiltration (Figure 3). Staining for fungus and bacteria detection was negative. The diagnosis of pompholyx was made, and the patient was treated with topical clobetasol 0.05% cream twice daily, for 15 days.

Pompholyx can be associated with or present manifestations of various dermatoses such as atopic dermatitis, contact dermatitis, adverse drug eruption, Id reactions, and HIV infection [1]. Unilateral pompholyx can be challenging to diagnose and needs to be distinguished from tinea pedis, bullous impetigo, herpes zoster, fixed drug eruption, and friction blister as all of them can produce itchy vesiculobullous lesions [1]. Pompholyx is known to be associated with hyperhidrosis and factors that promote an increase in sweating. It is exacerbated by hot and humid environments, stress, smoking, and the use of occlusive gloves. The association of pompholyx with increased sweating is further supported by the acral location of pompholyx, which has the highest concentration of sweat glands, increased perspiration volume in patients with pompholyx, and iontophoresis as a modality of therapy [1]. In our case, the unilateral localization of pompholyx was associated with an ipsilateral upper limb paresis and anterior horn disease. A similar case of unilateral pompholyx has been reported in association with amyotrophic lateral sclerosis (ALS). The authors attributed the disease-associated sympathetic overactivity to be responsible for the increased sweating and development of pompholyx [2]. Various cutaneous changes have been described in the paralytic limb, such as edema, reduced minimum erythema dose, increased tanning, and reduced sebum secretion. In the index case, the altered autonomic nervous function is possibly responsible for the unilateral localization of the pompholyx, as evidenced by increased sweating [3]. The sympathetic system’s role is further backed up by the resolution of the right-sided eczema and hyperhidrosis with the persistence of contralateral hyperhidrosis and eczema, following right-sided sympathectomy [4]. It is postulated that the high concentration of cytokines and proteases detected in the sweat stimulate inflammation and spongiosis, leading to the development of pompholyx [4].

## Conclusion

In conclusion, we reported a rare case of unilateral pompholyx in a patient with anterior horn disease that was localized to the ipsilateral side of the upper limb paresis.
